# Comparison of different anastomosis angles in radiocephalic fistula with modified functional end-to-side anastomosis

**DOI:** 10.1007/s11255-023-03608-y

**Published:** 2023-04-28

**Authors:** Wei Xu, Guoyuan Lu, Weigang Tang, Lifeng Gong, Jingkui Lu

**Affiliations:** 1 Department of Nephrology, The First Affiliated Hospital of Soochow University, Suzhou, 215006 Jiangsu China; 2 Department of Nephrology, Wujin Hospital Affiliated With Jiangsu University, Changzhou, 213000 Jiangsu China; 3 Department of Nephrology, The Wujin Clinical College of Xuzhou Medical University Changzhou, Changzhou, 213000 Jiangsu China

**Keywords:** Arteriovenous fistula, Functional end-to-side anastomosis, Anastomosis angle, Patency

## Abstract

**Objective:**

Functional vein end to arterial side (ETS) anastomosis uses vein side to arterial side anastomosis with distal vein ligation, which is different from traditional ETS anastomosis. To date, there are no studies concerning different anastomotic angles of fistula with functional ETS anastomosis. The purpose of the study was to analyze the clinical outcomes concerning different anastomotic angles of functional ETS anastomosis in radiocephalic fistula.

**Methods:**

Between January 2018 and December 2020, we performed a prospective cohort study concerning functional ETS anastomosis in radiocephalic fistula. According to vascular anatomy of patients, the anastomosis angles of fistula were designed at 30 ≤ angle ≤ 50°, 50 < angle ≤ 70°, and 135° smooth obtuse angle. The end points were the primary patency rate (PPR), the secondary patency rate (SPR) and the cumulative rate of reintervention (CRR) near anastomotic venous segment.

**Results:**

124 patients with functional ETS anastomosiss were enrolled in this study. Pearson *χ*^2^ test showed that the group of 135°anastomosis angle had the maximum distance between arteries and veins, and the group of 30–50°anastomosis angle had the minimum distance between arteries and veins (*P* < 0.01). 30–50°anastomosis angle had the highest PPR at 12 months (*P* = 0.03) and the lowest CRR near anastomotic venous segment at 3 months (*P* = 0.04) and 12 months (*P* = 0.01). There were no significant differences among different anastomosis angles concerning the SPR within 12 months (*P* > 0.05). Kaplan–Meier and log-rank analysis showed that 30–50°anastomosis had the highest PPR (*P* = 0.03) and the lowest CRR near anastomotic venous segment (*P* = 0.01). A multivariable Cox model showed anastomotic angle was an independent factor predictive of the PPR (*P* = 0.04) and the CRR near anastomotic venous segment (*P* = 0.03). 50–70°anastomosis angle was a risk factor of decreasing PPR (*P* = 0.03). 50–70° (*P* = 0.01) and 135° (*P* = 0.03) anastomosis angle were both obvious risk factors of increasing CRR near anastomotic venous segment.

**Conclusion:**

30–50°were the best anastomotic angles for functional ETS anastomosis, which had the highest PPR and lowest CRR near anastomotic venous segment.

## Introduction

Autogenous arteriovenous fistula (AVF) is the first choice for long-term hemodialysis vascular access [1, 2].The radiocephalic AVF at the wrist of the forearm is considered firstly in the establishment of AVF [3]. The common methods of AVF anastomosis used in patients with ESRD are vein end to arterial end (ETE), vein side to arterial side (STS), and vein end to arterial side (ETS) [4]. In clinical practice, ETS anastomosis is the most common method because of higher proximal venous flow, longer fistula survival and lesser long-term complications [5]. European Society for Vascular Surgery guidelines also recommend ETS anastomosis [6].

**Fig. 1:**
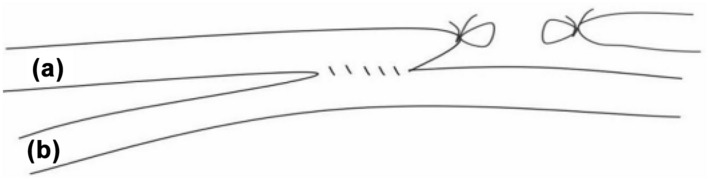
Functional ETS anastomosis uses side-to-side anastomosis with distal vein ligation. **a** Cephalic vein; **b** Radial artery

In recent years, some scholars reported a modified AVF anastomosis, which had good result [7]. This modified AVF anastomosis was named functional ETS anastomosis using side-to-side anastomosis with distal vein ligation, which achieved similar effects as those of ETS after STS anastomosis (Fig. 1). Our research group conducted a meta-analysis to compare the clinical outcomes between traditional and functional ETS anastomosis in radiocephalic fistula for dialysis access [8]. Our meta-analysis showed that functional ETS anastomosis had the advantages in easy operation, large anastomotic diameter, high surgical success rate, few complications, high long-term patency rate [8].

However, some surgical operators have questioned this AVF anastomosis design when the distance between the artery and vein is large. When the vein is far away from the artery, the functional ETS anastomosis is more likely to form an anastomotic angle of nearly 90°, resulting in angulation deformity. Anastomosis angle is an influential factor for failure of AVF. Computational models showed that the anastomotic angle affects the pattern of shear stress and blood flow from the anastomosis to its downstream. Hemodynamic disturbance and turbulence frequently lead frequently to the local development of intimal hyperplasia [9, 10]. The best anastomosis angles of traditional ETS anastomosis were different in the different studies. The study of Prouse showed the best anastomosis angles approached 60–70° [11]. The study of Lee showed the best anastomosis angle was 135° [12]. The study of Sadaghianloo showed the anastomotic angles of < 90°or ≥ 90° might not play a role in outcome of brachial-cephalic fistulas [13]. Van Canneyt concluded that an anastomotic angle between 30 and 45° were the best anastomosis angles [14]. The design of functional ETS anastomosis is not exactly the same as the traditional ETS anastomosis. To date, there are no studies on different anastomotic angles of fistula with functional ETS anastomosis. Therefore, our research group compared the clinical outcomes among the functional ETS anastomosis of different anastomosis angles in radiocephalic fistula at the wrist of the forearm. In particular, we discussed the optimal anastomotic angle of functional ETS anastomosis when the vein is far away from the artery. We hope our analysis will be useful to our peers.

## Methods

### Patients and design

This prospective cohort study included all radiocephalic fistulas created at Wujin Hospital Affiliated with Jiangsu University from January 1, 2018, to December 31, 2020. The inclusion criterias were as follow. Patients had the indications and conditions for establishing autogenous AVF according to KDOQI Clinical Practice Guideline for Vascular Access [15]. Radiocephalic AVF was established at the wrist of the forearm for the first time.

The exclusion criteria were as follows: (1) patient < 18 or > 75 years of age, (2) artery size < 2 mm, (3) vein size < 2 mm, (4) stenosis of the arterial system exist, (4) stenosis of the central vein, implantation materials of the central vein, (5) heart failure and other serious cardiovascular diseases, (6) cooperate with difficulty, (7) skin infection, (8) abnormal blood coagulation function.

### Surgical procedures and grouping

**Fig. 2:**
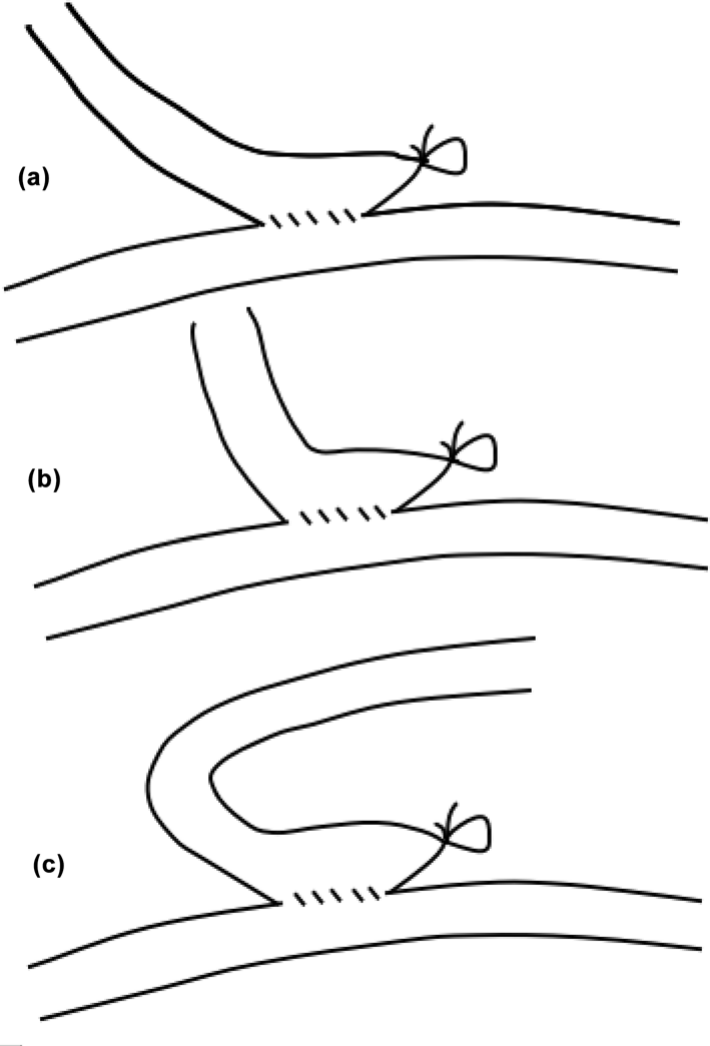
**A** Functional ETS anastomosis with 30–50° anastomosis angle; **B** Functional ETS anastomosis with 50–70° anastomosis angle; **C** Functional ETS anastomosis with 135° smooth obtuse angle

Before the operation, the sizes of arteries and veins were measured by ultrasound, the shape of arteries and veins was marked, and the distance between arteries and veins was measured. Surgical operations of the fistula were performed by a same experienced surgeon under local anesthesia. After separating the arteries and veins, the anastomosis angle was designed according to the vessel length, size, shape and especially distance of the vein from the artery. Anastomosis method was functional ETS anastomosis using side-to-side anastomosis with distal vein ligation. The length of anastomosis usually reached 10–12 mm. A tension-free anastomosis was performed with a continuous suture of 7–0 suture line. When the anastomosis was complete, the protractor was used to measure the anastomotic angle precisely. Grouping was according to AVF anastomosis angle, dividing into groups of 30 ≤ angle ≤ 50°, 50 < angle ≤ 70°, and 135°smooth obtuse angle (Fig. 2). The grouping was not random, but was based on vascular anatomy.

### Follow-up

Nephrologists track the access through clinical examinations and access flow monitoring monthly during hemodialysis. The first examination of ultrasound is performed at 4 weeks after surgery to measure vessel diameter and access flow and then every 2–3 months typically. If access shows sign of dysfunction, suspicious stenosis or thrombus, we use ultrasound or angiography to diagnose. Endovascular intervention and open surgery are used for treatment of access dysfunction, thrombus and stenosis. The follow-up time was 1 year.

### Outcomes and definitions

The end points were the primary patency rate(PPR) and the secondary patency rate(SPR). Primary patency is the interval from the time of access placement until any intervention designed to maintain or reestablish patency, access thrombosis, or the time of measurement of patency. Secondary or cumulative patency was calculated from the time of vascular access creation until permanent access failure, regardless of the number of procedures required to maintain access patency for dialysis [16]. The other end point was the cumulative rate of reintervention close to 4 cm vein of anastomotic segment [17, 18]. Chinese guideline defined access as functional when natural blood flow > 500 ml/min, the pump controlled blood flow during dialysis was greater than 200 ml/min maintained for 4 h [19].

### Statistical analysis

We performed the data analysis using spss 22 statistical software. Categorical variables were expressed as the number and percentage of patients. Differences between categorical variables were determined by the Pearson *χ*^2^ test. Continuous variables were expressed as the mean ± standard error. Differences between continuous variables were determined by the One-way analysis of variance. Kaplan–Meier analysis was used to calculate the curves of patency rate and the cumulative rate of reintervention near anastomotic venous segment, with curves compared using log-rank analysis. The Cox proportional hazards regression model was used to control potentially confounding factors. Values of *P* < 0.05 was considered statistically significant.

## Results

During the study period, 124 AVF fistulas with functional ETS anastomosiss were constructed in 124 patients. Anastomosis angles of 54 patients were 30–50°, anastomosis angles of 39 patients were 50–70°, and anastomosis angles of 31 patients were 135°smooth obtuse angles. 1 patient with 30–50° anastomosis angle was lost to follow-up within 3 months after surgery. 1 patient with 30–50° anastomosis angle and 1 patient with 50–70° anastomosis angle were lost to follow-up at 3–6 months after surgery. The baseline characteristics of these patients are listed in Table 1.The group of 135° anastomosis angle had the maximum distance between arteries and veins. The group of 30–50° anastomosis angle had the minimum distance between arteries and veins. There was significant difference among the groups of 30–50°,50–70° and 135° anastomosis angle concerning the distance between arteries and veins (*P* < 0.01) (Table 1).

**Table 1:** Patient characteristics for functional ETS anastomosis

Variable	30–50° angle (*n* = 54), No. (%) or mean ± SE	50–70° angle (*n* = 39), No. (%) or mean ± SE	135° angle (*n* = 31), No. (%) or mean ± SE	*P*
Age (years)	56.2 ± 11.2	52.3 ± 13.1	54.2 ± 13.6	0.33
Gender				0.43
Female	30 (55.6)	17 (43.6)	18 (58.1)	
Male	24 (44.4)	22 (56.4)	13 (41.9)	
Scr (μmol/L)	650.8 ± 130.9	698.6 ± 184.7	659.5 ± 208.9	0.39
HB (g/L)	92.5 ± 13.0	89.8 ± 10.1	91.6 ± 11.2	0.56
PLT (10*9/L)	187.5 ± 55.2	180.6 ± 56.1	177.4 ± 55.2	0.69
Vein size (mm)	2.24 ± 0.22	2.28 ± 0.28	2.30 ± 0.30	0.57
Artery size (mm)	2.39 ± 0.23	2.35 ± 0.32	2.44 ± 0.26	0.37
The distance between artery and vein (cm)	1.3 ± 0.4	2.9 ± 0.4	3.3 ± 0.3	< 0.01
Cause of renal disease				0.98
Diabetes	21 (38.9)	15 (38.5)	12 (35.5)	
Hypertension	8 (14.8)	5 (12.8)	2 (6.5)	
Glomerulonephritis	13 (24.1)	11 (28.2)	9 (29.0)	
Polycystic kidney disease	4 (7.4)	2 (5.1)	2 (6.5)	
Systemic lupus erythematosus	2 (3.7)	1 (2.6)	2 (6.5)	
ANCA associated vasculitis	1 (1.9)	0 (0)	1 (3.2)	
Hepatorenal syndrome	0 (0)	1 (2.6)	0 (0)	
Unknown/undetermined	5 (9.3)	4 (10.3)	3 (9.7)	
Comorbidity				
Smoking	24 (44.4)	13 (33.3)	12 (38.7)	0.55
Diabetes	27 (50.0)	19 (48.7)	14 (45.2)	0.94
Hypertension	40 (74.1)	29 (74.4)	20 (64.5)	0.58

In terms of functional ETS anastomosis, there was significant difference among the groups of different anastomosis angles concerning the PPR at 12 months (*P* = 0.03). However, in the pairwise comparison of different anastomosis angle groups concerning the PPR at 12 months, only the PPR of 30–50° anastomosis angle was statistically higher than 50–70° anastomosis angle (*P* < 0.05). There were no significant differences among the groups of different anastomosis angles concerning the 3 months PPR (*P* = 0.21), 6 months PPR (*P* = 0.31), 3 months SPR (*P* = 0.87), 6 months SPR (*P* = 0.92) and 12 months SPR (*P* = 0.65) (Table 2).

**Table 2:** Patency rates and cumulative rate of reintervention near anastomotic segment for functional ETS anastomosis with differrnt anastomosis angles

Variable	30–50° angle (*n* = 54)	50–70° angle (*n* = 39)	135° angle (*n* = 31)	*P*
Primary patency, %				
At 3 months	92.5	82.1	80.6	0.21
At 6 months	86.5	73.7	77.4	0.31
At 12 months	82.7^a^	57.9^a^	64.5	0.03
Secondary patency, %				
At 3 months	96.2	94.9	93.5	0.87
At 6 months	92.3	89.5	90.3	0.92
At 12 months	90.4	84.2	83.9	0.65
Cumulative rate of reintervention near anastomotic segment				
At 3 months	1.9^b^	15.8^b^	12.9	0.04
At 6 months	5.8	18.4	16.1	0.12
At 12 months	9.6^c^	34.2^c^	25.8	0.01

A *P* value of less than 0.05 indicates unequal or incomplete equivalence of the outcomes among the three groups of different anastomotic angles. The outcomes with identical superscripts indicate statistical differences in pairwise comparisons

There was significant difference among the groups of different anastomosis angles concerning the cumulative rate of reintervention (CRR) close to 4 cm vein of anastomotic segment at 3 months (*P* = 0.04) and 12 months (*P* = 0.01). In the pairwise comparison of different anastomosis angle groups concerning the CRR close to anastomotic venous segment at 3 months, only the CRR of 30–50° anastomosis angle was statistically lower than 50–70° anastomosis angle (*P* < 0.05). In the pairwise comparison of different anastomosis angle groups concerning the CRR close to anastomotic venous segment at 12 months, only the CRR of 30–50°anastomosis angle was statistically lower than 50–70° anastomosis angle (*P* < 0.05). There were no significant differences among the groups of different anastomosis angles concerning the CRR close to anastomotic venous segment at 6 months (*P* = 0.12) (Table 2).

**Fig. 3:**
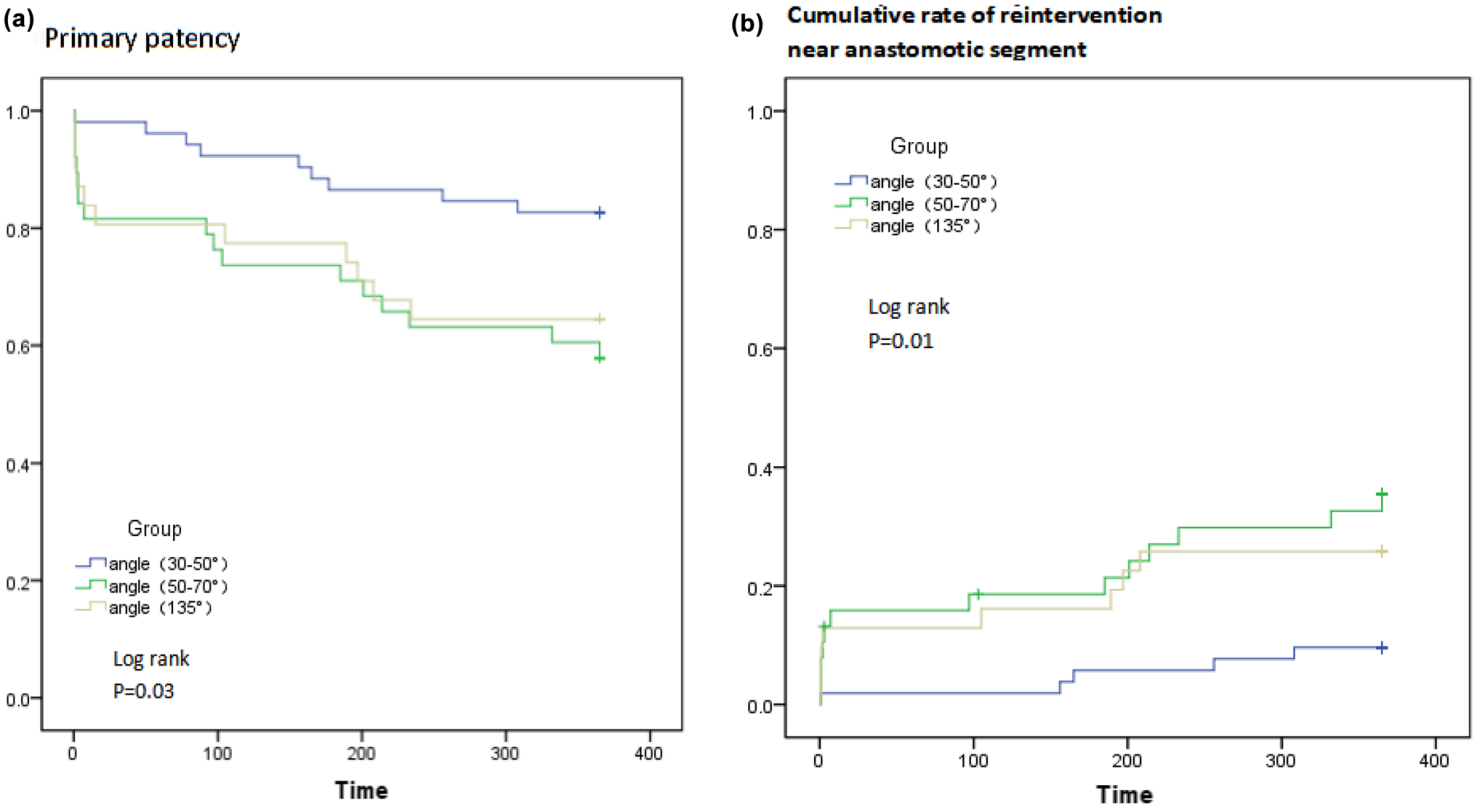
Kaplan–Meier and log-rank analysis of differrnt anastomosis angle concerning **A** primary patency and **B** cumulative rate of reintervention close to anastomotic venous segment in functional ETS anastomosis

Kaplan–Meier and log-rank analysis showed that 30–50° anastomosis angles had highest PPR (*P* = 0.03) and lowest CRR close to anastomotic venous segment (*P* = 0.01) (Fig. 3). A multivariable Cox model was performed to determine factors predictive of the PPR and the CRR near anastomotic segment (Table 3). Anastomotic angle was an independent factor predictive of the PPR (*P* = 0.04) and the CRR close to anastomotic venous segment (*P* = 0.03) in patients with a functional ETS anastomosis. 50–70° anastomosis angle was a risk factor of decreasing primary patency rate (*P* = 0.03,). 50–70° (*P* = 0.01) and 135° (*P* = 0.03) anastomosis angle were both obvious risk factors of increasing CCR close to anastomotic venous segment. In this study, venous size was a potentially factors for the PPR (*P* = 0.02), arterial sizes was a potentially factors for the CRR close to anastomotic venous segment (*P* = 0.04) (Table 3).

**Table 3:** Multivariable Cox analysis for primary patency rate and cumulative rate of reintervention near anastomotic segment in functional ETS anastomosis

Variable	HR	95% CI	*P*
Primary patency			
Angle			0.04
30–50°	1.00 (ref)		
50–70°	2.81	1.22–6.43	0.03
135°	2.60	1.07–6.29	0.85
Vein size	0.10	0.01–0.73	0.02
Artery size	0.16	0.02–1.12	0.07
Cumulative rate of reintervention near anastomotic segment			
Angle			0.03
30–50°	1.00 (ref)		
50–70°	3.83	1.34–11.01	0.01
135°	3.41	1.11–10.45	0.03
Vein size (mm)	0.32	0.04–2.10	0.23
Artery size (mm)	0.10	0.01–0.89	0.04

Concerning setting the dummy variable of anastomosis angle, the angle of 30–50° was used as a reference

## Discussion

Radiocephalic AVF with functional ETS anastomosis had the advantages in easy operation, large anastomotic diameter, high surgical success rate, few complications, high long-term patency rate compared traditional ETS anastomosis, which were shown in our previous meta-analysis [8]. The anastomotic angle influences the blood flow and shear stress near the anastomosis, with areas of too low, too high, or turbulent shear stress frequently leading to localized development of intimal hyperplasia [20, 21]. Therefore, anastomosis angle is an influential factor for AVF concerning the patency rate and the cumulative rate of reintervention close to anastomotic venous segment. If we can find the best anastomotic angle for functional ETS anastomosis, patients will have greater long-term benefits. Our study showed 30–50° were the best anastomotic angles for functional ETS anastomosis.

Functional ETS anastomosis uses side-to-side anastomosis with distal vein ligation. In functional ETS anastomosis, the long axis of the vein is parallel to the artery at the anastomotic stoma, then away from the anastomosis the long axis of the vein forms an angle with the artery, which is similar to that of STS anastomosis. In our study, if the vein is far away from the artery, the anastomotic angle becomes large. When the vein exits the anastomosis stoma at 90° anastomotic angle using functional ETS anastomosis, the vein will show obvious angulation deformity and possibly narrow the diameter of the vein, which is different from traditional ETS anastomosis. Several studies have shown that 30–45°, 60–70°, 135°in traditional ETS anastomosis might be good anastomotic angle analyzed by the flow pattern in models of AVF using three-dimensional grids and computational modeling [11–14]. Therefore, in our study of functional ETS anastomosis, if the artery and vein were far apart, we tried to form an acute angle of less than 70° or a smooth obtuse angle of 135° by fully separating the vein.

Our study showed 30–50° of anastomotic angles had highest primary patency and lowest cumulative rate of reintervention close to anastomotic venous segment. The possible causes are that 50–70° anastomotic angles in functional ETS anastomosis still increase the risk of angulation deformity compared with 30–50°. Angulation deformity may lead low shear stress and turbulent shear stress, which are related with local intimal hyperplasia [21]. The study of Canneyt showed that an anastomotic angle between 30° and 45° would create fewer low-flow zones [14]. The study of Lee showed that 135° anastomotic angle had suitable shear stress analyzed by computational fluid dynamic, which helped reduce the AVF failure [12]. However, the anastomotic angle of 135° smooth obtuse did not showed advantage in our study. In the actual operation, 135°anastomotic angle is possible to become unsmooth or angulation deformity, due to the softness of the veins, the obstruction or traction of the surrounding soft tissues and the pull of closing the skin incision. We propose an idea of whether the traditional ETS anastomosis is better when the distance between the artery and vein is far.

There were some limitations in our study. This study included a relatively small number of patients in each group. There are many factors affecting the patency of AVF, and it was difficult to control all confounding factors in our study. The anastomotic angle was measured in two dimensions and did not capture in the three-dimensional geometry. Besides, we hope that the three-dimensional grids and computational models can be used to analyze the blood fluid dynamic in fistula with functional ETS anastomosis.

## Conclusions

Our study showed 30–50° were the best anastomotic angles for functional ETS anastomosis, which had highest primary patency and lowest cumulative rate of reintervention close to anastomotic venous segment. To further confirm the conclusion, more large multicenter trials and the computational models analysis comparing different anastomotic angles in fistula with functional ETS anastomosis are necessary.
